# A contact detection algorithm for polyhedrons based on improved common-plane concept

**DOI:** 10.1038/s41598-025-23605-8

**Published:** 2025-11-14

**Authors:** Mingqing Liu

**Affiliations:** 1 College of Architecture and Energy Engineering, Wenzhou University of Technology, Wenzhou, 325035 China; 2 Wenzhou Key Laboratory of Intelligent Lifeline Protection and Emergency Technology for Resilient City, Wenzhou University of Technology, Wenzhou, 325035 China

**Keywords:** DEM, Polyhedrons, Contact detection, Common-plane concept, Engineering, Mathematics and computing

## Abstract

To address the inaccuracies in contact point calculation and low computational efficiency of traditional common-plane methods in complex contact scenarios, this study proposes an improved contact detection algorithm based on the common-plane concept. By defining a distance function between block elements and the common plane, optimizing the contact point update strategy, and introducing a dual-condition-controlled iterative termination mechanism, the algorithm significantly enhances computational precision for edge-edge, edge-face, and other contact types. Validation using CAD models demonstrates that the coordinate error of contact points is less than 5%, the normal angle deviation is below 0.5°, and the contact depth error is controlled within 1%. Discrete Element Method (DEM) simulations of polyhedron random packing processes reveal that the improved algorithm increases computational efficiency by over 10% compared to the traditional common-plane method, while reducing the maximum contact depth error by 47.4%. Experimental and simulated particle packing morphologies and trajectories show high consistency, confirming the algorithm’s reliability and engineering applicability.

## Introduction

The Discrete Element Method (DEM) originated from studies on the mechanical behavior of granular materials and particle media^1^. In the 1970s, Cundall and Strack first introduced this method^2^ to simulate interactions between particles, aiming to investigate the mechanical properties of soils, sands, and granular materials^3,4^. Unlike traditional continuum models, DEM treats materials as collections of discrete particles, enabling realistic modeling of interparticle interactions and motion^5^. With advancements in computational technology, DEM has evolved into a powerful numerical tool widely applied in civil engineering, mining, agriculture, and materials science^6–9^. Its advantages lie in handling complex interparticle interactions, nonlinear behaviors, and large deformations. Furthermore, DEM can simulate random particle packing processes, revealing macroscopic mechanical behaviors of granular materials and providing critical insights for engineering design and analysis^10–12^.

Within the DEM framework, particle motion and interactions are governed by physical models, where contact detection serves as the foundation. Contact detection not only initiates the mechanical behavior between particles but also determines the physical realism of simulations. Accurate identification of contact states and force calculations ensures reliable representation of interparticle interactions, directly influencing simulation outcomes^13–15^.

Contact detection holds a central role in DEM, affecting both precision and computational efficiency. Inaccuracies in contact detection can lead to erroneous force calculations, distorting the mechanical response of granular materials. This is particularly crucial in geotechnical engineering simulations, where soil and rock behaviors are heavily influenced by interparticle interactions. Thus, developing efficient and accurate contact detection algorithms is essential for advancing DEM applications in engineering^16,17^.

In geotechnical DEM simulations, block elements offer a more realistic representation of actual conditions^18^, especially for complex structures. Block elements enable explicit modeling of components such as rocks, soils, and voids. Unlike continuum models, block elements capture interactions, contact forces, and their evolution during deformation. This approach is particularly suited for simulating phenomena like settlement, failure, and landslides under external loads^19,20^. By adjusting contact models within block elements, researchers can simulate nonlinear, plastic, and fracture behaviors, achieving accurate descriptions of geomechanical properties^21–23^.

Despite the advantages of block elements, existing contact detection algorithms face challenges:

(1) **Low computational efficiency**: Exponential growth in computational load for large-scale systems.

(2) **Insufficient accuracy**: Deviations in contact point and normal calculations for non-point contact types (e.g., edge-edge, face-face).

Cundall’s Common Plane Method (CPM) simplifies contact detection by constructing a common plane but suffers from iterative inefficiency and single termination conditions, leading to high computational costs and potential deadlocks in complex contact scenarios. Other prevalent contact detection algorithms include the Axis-Aligned Bounding Box (AABB) algorithm, the Oriented Bounding Box (OBB) algorithm, and the Gilbert–Johnson–Keerthi (GJK) algorithm combined with the Expanding Polytope Algorithm (EPA). While AABB and OBB are highly efficient for broad-phase collision detection due to their simple volume overlap checks, they are primarily used as a preliminary filtering step and require subsequent precise detection algorithms (like CPM or GJK-EPA) to resolve the exact contact features for polyhedrons. The GJK-EPA algorithm is capable of handling convex polyhedrons but can be computationally expensive and complex to implement for all feature pairs (e.g., edge-edge, face-face). The proposed improved CPM algorithm focuses on the precise detection phase, offering a unified and efficient framework for determining the exact contact parameters between polyhedrons. Its computational efficiency, particularly for the edge-edge and face-face contacts common in DEM simulations of granular materials, is achieved through the optimized update strategy and dual-condition termination mechanism, reducing the number of iterations required compared to a naive implementation of other methods for the same precision. To address these issues, this paper proposes an improved common-plane algorithm incorporating distance functions, optimized contact point updates, and dual-condition termination mechanisms for efficient and precise contact detection.

## Improved common-plane method

### Limitations of traditional common-plane method

**Fig. 1:**
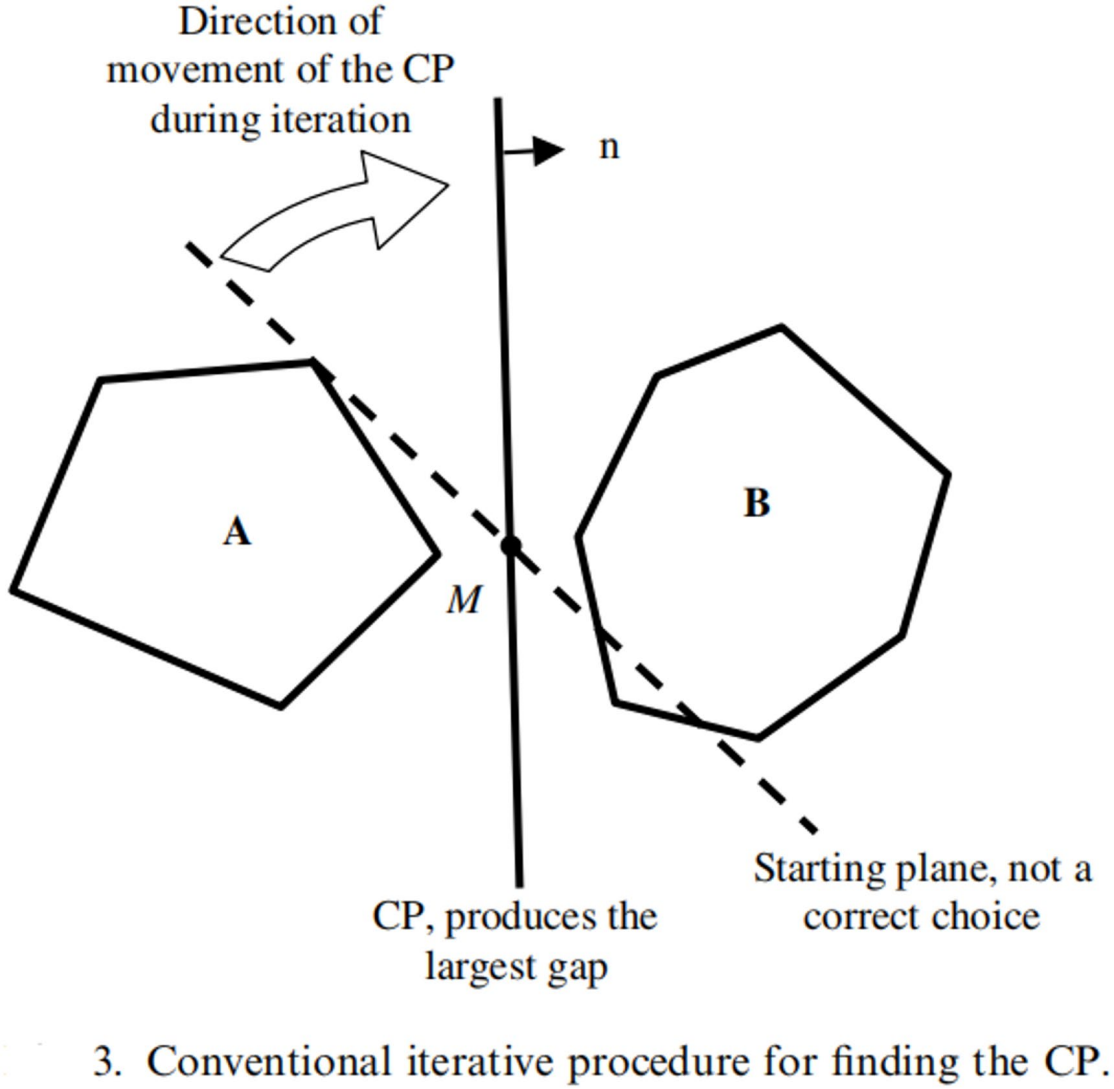
Conventional iterative procedure for finding the CP.

**Fig. 2:**
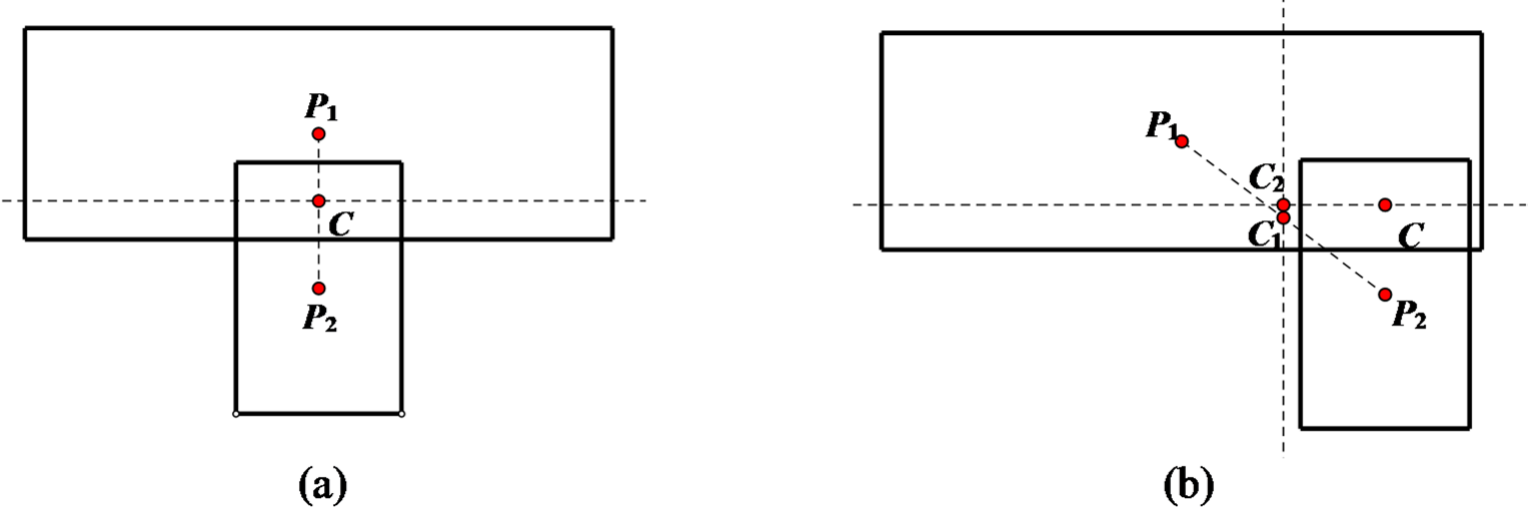
Solution of contact point in two-dimensional case. (**a**) Edge-to-edge contact, (with the line connecting the centroids perpendicular to the common plane), (**b**) Edge-to-edge contact, (with the line connecting the centroids not perpendicular to the common plane)

The traditional CPM identifies contact states by searching for the plane of maximum separation between two particles (Fig. 1). Key limitations include:

(1) **Low iterative efficiency**: Multiple plane rotations per contact detection; iteration counts surge with poor initial guesses.

(2) **Contact point errors** In edge-edge contacts, contact points deviate from true positions when centroid lines are non-perpendicular to the common plane (Fig. 2).

(3) **Termination flaws** Prone to infinite loops in hexahedral contacts.

### Algorithm improvements

#### Distance function definition

In the common plane method, the calculation of the distance from a point to a plane is frequently employed. The introduction of a distance function facilitates the calculation of such distances and enhances computational efficiency. The so-called distance function involves inserting a plane (or a straight line) between two block elements and calculating the distances from each block element to the plane (or line) separately. The expressions for calculating the distances from the block elements to the plane (or line) constitute the distance function. Assuming that a point **p** on the plane (or line) and the normal vector **n** of the plane (or line) are known, the distance functions from the block elements to the plane (or line) are presented in Table 1.

**Table 1:** Distance functions for block elements.

Shape element	Distance function	Meaning of characters
Polygon	${f_d}=\hbox{max} ({\mathbf{v}_i} - {\mathbf{p}_l}) \cdot {\mathbf{n}_l}$	“**v**_i_” represents the i-th vertex of a block (or polyhedral) element.
polyhedron		

**Fig. 3:**
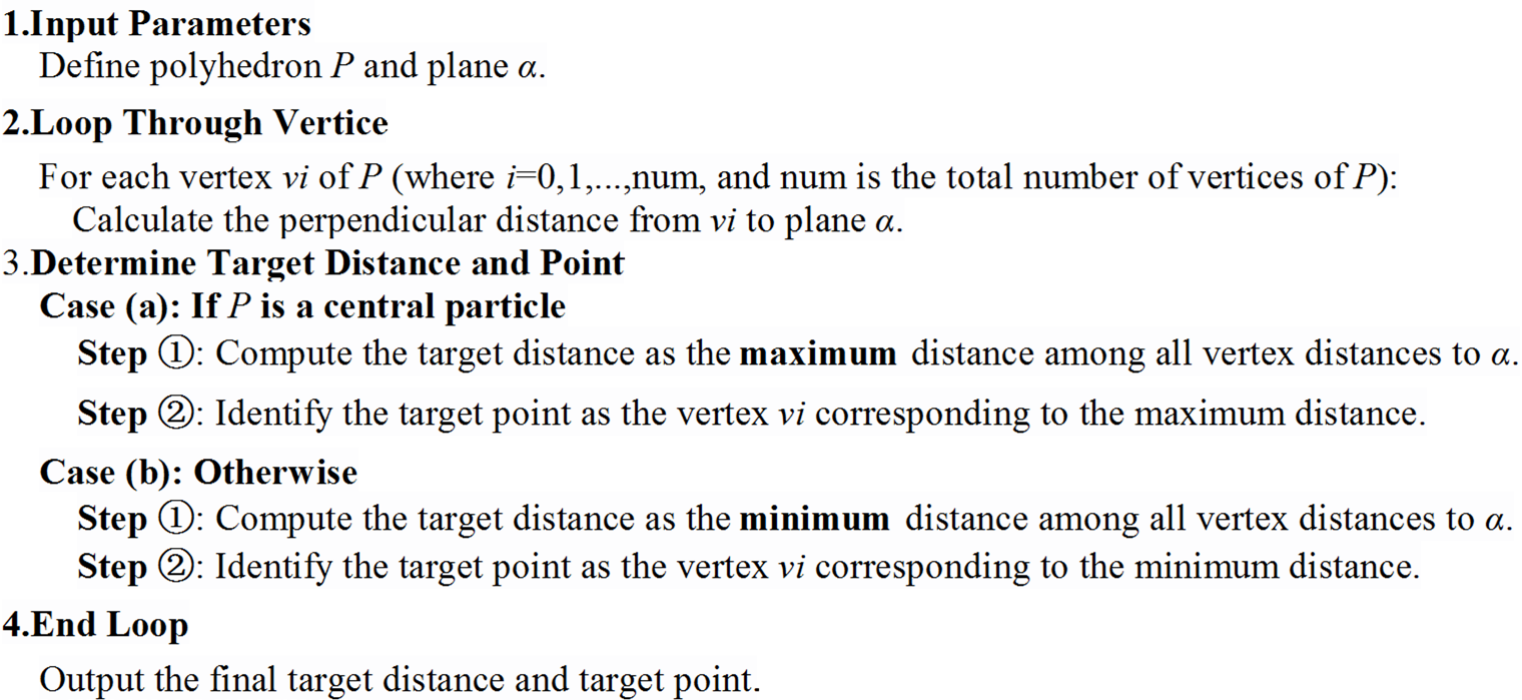
Distance function from polyhedral element P to plane α.

Taking the polyhedral element as an example, the specific process of its distance function to the plane is shown in Fig. 3.

#### Contact point update optimization

**(1) Polygon-polygon contacts**.

The algorithm for updating contact points between polygons can be categorized into three types of contact scenarios: point-point, point-edge, and edge-edge. Below is an elaboration for each case.

① Point-Point Contact.

In this scenario, the algorithm for updating contact points is consistent with the method for determining common planes, and thus, it will not be elaborated further here.

② Point-Edge Contact.

In this case, the distance function is used to calculate the shortest distance from each polygon to the common line (or edge) and to determine the corresponding closest points. The contact points can be computed using formulas (1) and (2).

**Fig. 4:**
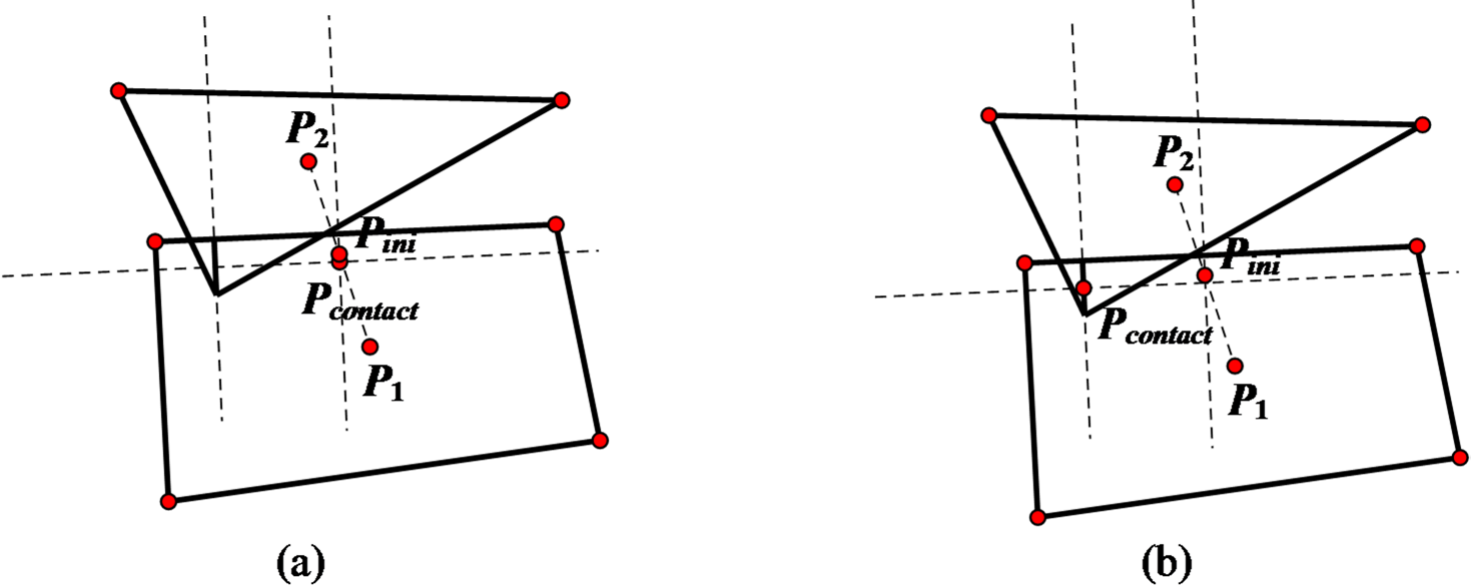
Comparison of updated contact point positions in point-edge contact scenarios. (**a**) Pre-improvement, (**b**) Post-improvement.

A comparison diagram showing the positions of updated contact points before and after improvement is presented in Fig. 4. From this diagram, it can be observed that the updated positions of contact points after improvement are more accurate.

1 $${\mathbf{P}_{contact\,}}={\mathbf{P}_1}+0.5gap\mathbf{n}$$

Otherwise, the contact point is determined as

2 $${\mathbf{P}_{contact\,}}={\mathbf{P}_2} - 0.5gap\mathbf{n}$$

In the formula, **P**_**1**_ and **P**_**2**_ are the nearest points on the two polygons to the common line, respectively. *gap* represents the contact depth, and **n** is the normal vector of the common line.

③ Edge-Edge Contact.

**Fig. 5:**
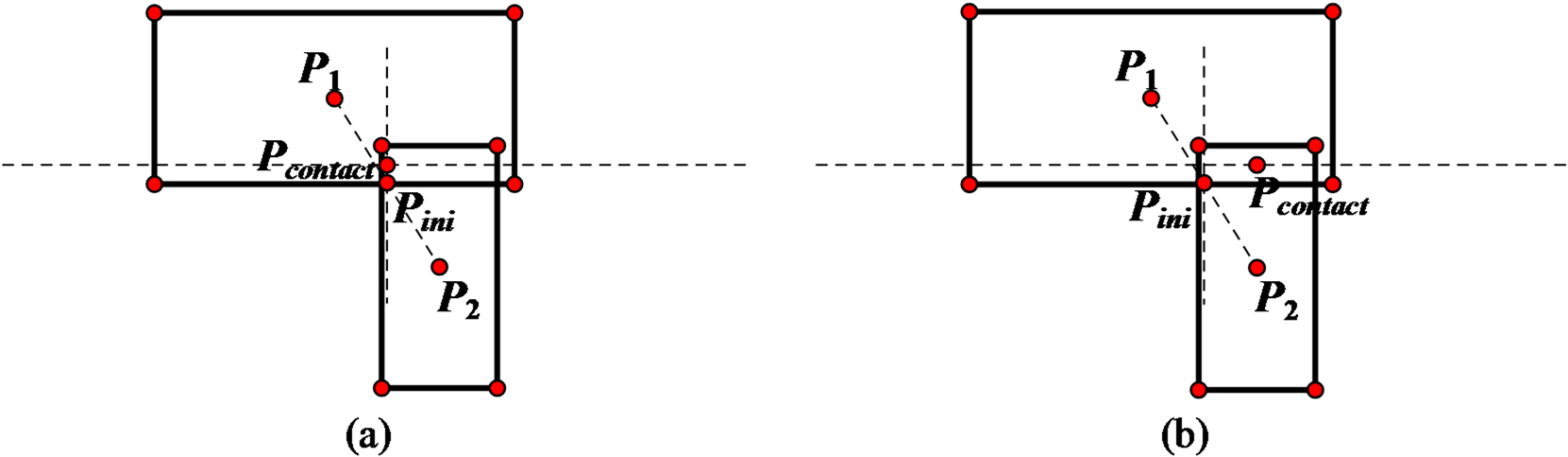
Comparison of updated contact point positions in edge-edge contact scenarios. (**a**) Pre-improvement, (**b**) Post-improvement.

In this scenario, the two mutually contacting edges, denoted as *edge1* and *edge2*, are first identified. Subsequently, these two edges are projected onto a common line, resulting in two projected edges, *edge11* and *edge22*. The number of intersection points between *edge11* and *edge22* is then determined. If there is exactly one intersection point, that point is designated as the desired contact point. If there are two or more intersection points, the average of these intersection points is taken as the desired contact point. A comparison diagram illustrating the updated contact positions before and after the improvement is shown in Fig. 5. From this diagram, it can be observed that the improved algorithm ensures that the updated contact point lies within the contact region and is closer to the centroid of that region.

**(2) Polyhedron-polyhedron contacts**.

The update algorithm for polyhedron-polyhedron contact points can be explained based on six types of contact scenarios: point-point, point-edge, point-face, edge-edge, edge-face, and face-face.

① Point-point contact.

**Fig. 6:**
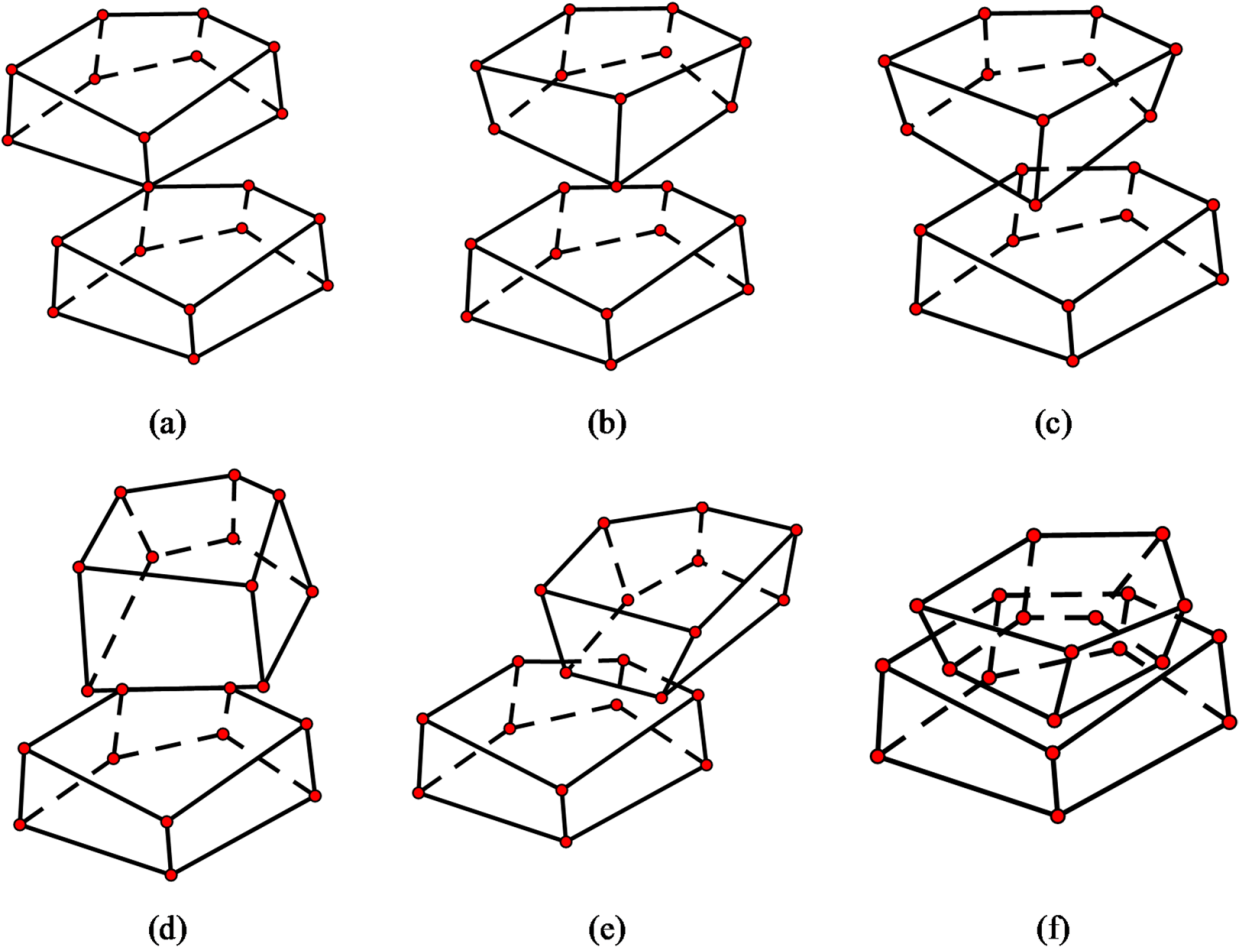
Six cases of polyhedron-polyhedron contact. (**a**) Point-point, (**b**) point-edge, (**c**) point-face, (**d**) edge-edge, (**e**) edge-face, (**f**) face-face.

As illustrated in Fig. 6(a), in this scenario, the nearest vertex on polyhedron 1 corresponding to the minimum distance to the common plane is **P**_**1**_, and the nearest vertex on polyhedron 2 corresponding to the minimum distance to the common plane is **P**_**2**_. Thus, the contact point is

3 $${\mathbf{P}_{contact\,}}=0.5({\mathbf{P}_1}+{\mathbf{P}_2})$$

② Point-edge contact.

Point-edge contact is illustrated in Fig. 6(b). In this scenario, assume that a point on polyhedron 1 is in contact with an edge on polyhedron 2. The nearest vertex on polyhedron 1 corresponding to the minimum distance to the common plane is **P**_**1**_, the normal vector of the common plane is **n**, and the clearance value is gap. If polyhedron 1 is the core particle, then the contact point is

4 $${\mathbf{P}_{contact\,}}={\mathbf{P}_1}+0.5gap\mathbf{n}$$

Otherwise, the contact point is

5 $${\mathbf{P}_{contact\,}}={\mathbf{P}_1} - 0.5gap\mathbf{n}$$

When assuming the case where an edge on polyhedron 1 is in contact with a point on polyhedron 2, the nearest vertex on polyhedron 2 corresponding to the minimum distance to the common plane is **P**_**2**_. To obtain the target contact point, simply replace **P**_**1**_ with **P**_**2**_ in the aforementioned formula.

③ Point-face contact.

As shown in Fig. 6(c), in this scenario, the method for calculating the contact point is the same as in the point-edge contact case. The contact point can be determined using formulas (4) and (5).

④ Edge-edge contact.

As illustrated in Fig. 6(d), in this scenario, the general equation of the common plane is first established. Since the reference point on the common plane is **F** and the normal vector is **n**, the general equation of the common plane is as shown in formula (6).

6 $$Ax+By+Cz+D=0$$

wherein, $$A = {x_n},B = {y_n},C = {z_n},D = - {x_n}{x_F} - {y_n}{y_F} - {z_n}{z_F}$$.

By utilizing the general equation of the common plane, the edges of the contacting polyhedron 1 and polyhedron 2 are projected onto the common plane, resulting in projected line segments *l*_1_ and *l*_2_, respectively. Subsequently, employing the normal vector of the common plane, a projection plane is generated. Using this projection plane, the line segments *l*_1_ and *l*_2_ are transformed into two-dimensional line segments *l*’_1_ and *l*’_2_, with their corresponding endpoints being **E**_**11**_, **E**_**12**_ and **E**_**21**_, **E**_**22**_, respectively. Following this, the positional relationship between the two two-dimensional line segments *l*’_1_ and *l*’_2_ is determined, and their intersection points and the number of such points are calculated, thereby computing the two-dimensional contact points.

If the number of intersection points between *l*’_1_ and *l*’_2_ is 1, denoted as **I**, then the two-dimensional contact point is

7 $${\mathbf{P}_{contact\,2D}}=\mathbf{I}$$

If the number of intersection points between *l*’_1_ and *l*’_2_ is greater than 1, with the intersection points being **I**_i_(*i* = 1,2…), then the two-dimensional contact point is

8 $${\mathbf{P}_{contact\,2D}}=({\mathbf{I}_1}+{\mathbf{I}_2}+ \cdots +{\mathbf{I}_i})/i$$

After the aforementioned process, the two-dimensional contact points can be obtained. Then, by utilizing the projection plane (plane) and the common plane, a transformation from two dimensions to three dimensions can be performed to yield the target contact point.

⑤ Edge-Face Contact.

As shown in Fig. 6(e), this case assumes contact between an edge of polyhedron 1 and a face of polyhedron 2.

First, establish the general equation of the common plane. Given reference point **F** and normal vector **n** on the common plane, the general plane equation is expressed as Eq. (6).

Project the contact edge of polyhedron 1 onto the common plane using its general equation. Then, generate a projection plane utilizing the normal vector of the common plane. Convert the line segment projected onto the common plane into a 2D line segment *l* using this projection plane, with endpoints **E**’_1_ and **E**’_2_. Similarly, convert the contact face of polyhedron 2 into a 2D contact face *p* using the projection plane, with corresponding points **Q**_*i*_(*i* = 1,2,…) on the face. Subsequently, determine the positional relationship between 2D line segment *l* and 2D contact face *p*, find their intersection points and the number of intersections, thereby calculating the 2D contact points.

**Fig. 7:**
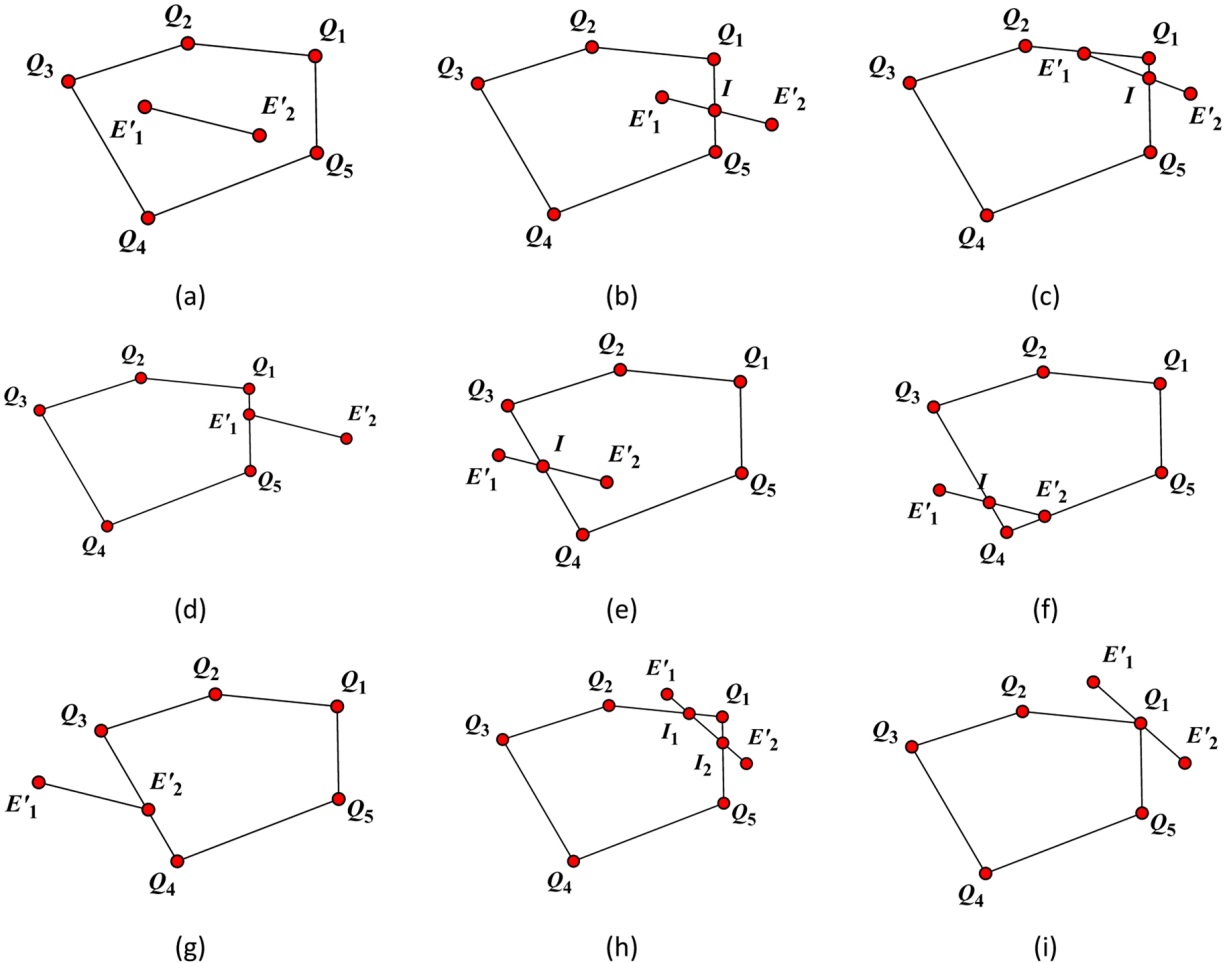
Position relationship between line segment and polygon.

As shown in Fig. 7, suppose the 2D plane is the plane on which the pentagon is located.

i. Point **E**’_1_ lies on or within the 2D plane.

If Point **E**’_2_ also lies on or within the same 2D plane, as illustrated in Fig. 7(a), then the 2D contact point is defined as follows:

9 $${\mathbf{P}_{contact\,2D}}=0.5({\mathbf{E}^{\prime}_1}+{\mathbf{E}^{\prime}_2})$$

If Point **E**’_2_ is located outside the 2D plane, and in the scenarios illustrated in Fig. 7b, c where the intersection point is labeled **I**, then the 2D contact point is defined by

10 $${\mathbf{P}_{contact\,2D}}=0.5({\mathbf{E}^{\prime}_1}+\mathbf{I})$$

In the case of Fig. 7(d), the 2D contact point is determined by

11 $${\mathbf{P}_{contact\,2D}}={\mathbf{E}^{\prime}_1}$$

ii. Point **E**’_1_ lies outside the 2D plane.

If point **E**’_2_ lies on or inside the 2D plane, and the intersection point is **I** as shown in Fig. 7(e) and (f), then the 2D contact point is

12 $${\mathbf{P}_{contact\,2D}}=0.5({\mathbf{E}^{\prime}_2}+\mathbf{I})$$

In the case of Fig. 7g, the 2D point is

13 $${\mathbf{P}_{contact\,2D}}={\mathbf{E}^{\prime}_2}$$

If point **E**’_2_ lies outside the 2D plane, when it is the case of Fig. 7(h), the intersection points are **I**_1_ and **I**_2_, then the 2D contact point is

14 $${\mathbf{P}_{contact\,2D}}=0.5({\mathbf{I}_1}+{\mathbf{I}_2})$$

In the case of Fig. 7(i), the 2D point is

15 $${\mathbf{P}_{contact\,2D}}={\mathbf{Q}_1}$$

Through the above process, the 2D contact point is obtained. Then, using the projection plane and the common plane, the target contact point can be obtained through the 2D to 3D conversion.

When assuming that the contact is between a face of polyhedron 1 and a side of polyhedron 2, the target contact point can be obtained by replacing the side of polyhedron 1 with the face of polyhedron 1 and replacing the side of polyhedron 2 with the face of polyhedron 2 in the above process and performing the same calculation.

⑥ Face-face Contact.

As shown in Fig. 7(g), in this case, firstly, a projection plane is generated using the normal vector **n** of the common plane. Then, using this projection plane, the two faces of polyhedron 1 and polyhedron 2 that are in contact with each other are converted into 2D contact faces *p*1 and *p*2, and the corresponding vertices are converted into **P**_*i*_(*i* = 1,2,…) and **Q**_i_(_i_=1,2,…).

**Fig. 8:**
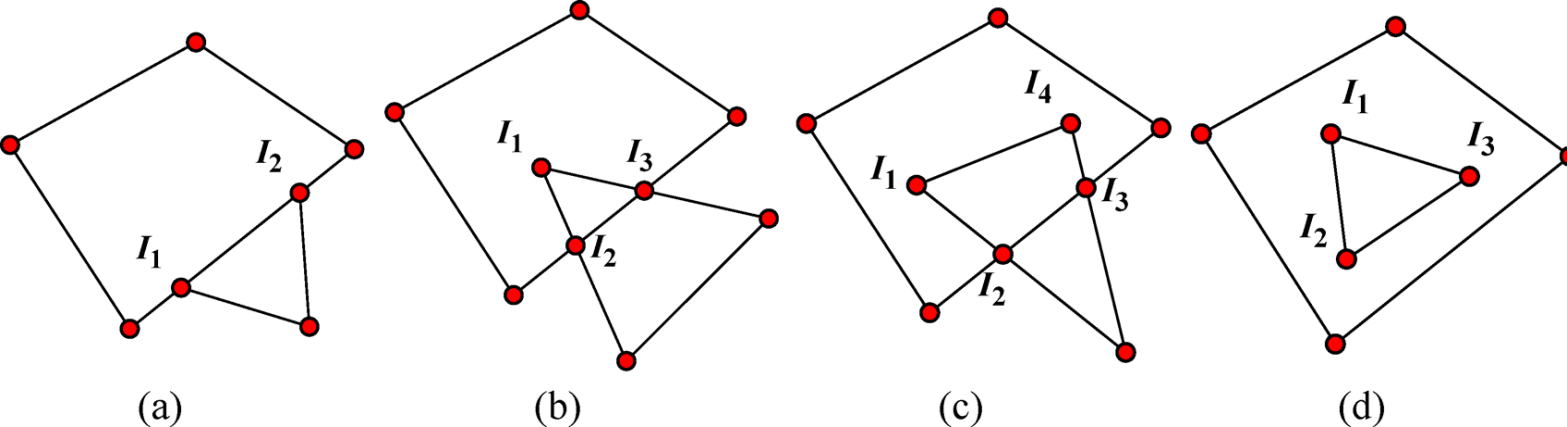
Quadrilateral-triangle contact.

If the number of intersection points is 2, as shown in Fig. 8(a), the intersection points are **I**_1_ and **I**_2_, then the 2D contact point is

16 $${\mathbf{P}_{contact\,2D}}=0.5({\mathbf{I}_1}+{\mathbf{I}_2})$$

If the number of intersection points is greater than 2, as shown in Fig. 8(b), (c) and (d), the intersection points are **I**_*i*_(*i* = 1,2,…), then the 2D contact point is

17 $${\mathbf{P}_{contact\,2D}}=({\mathbf{I}_1}+{\mathbf{I}_2}+ \cdots +{\mathbf{I}_i})/i$$

Through the above process, the 2D contact point can be obtained. Using the projection plane and the common plane, the 2D is transformed into 3D, and the target contact point can be obtained.

**Fig. 9:**
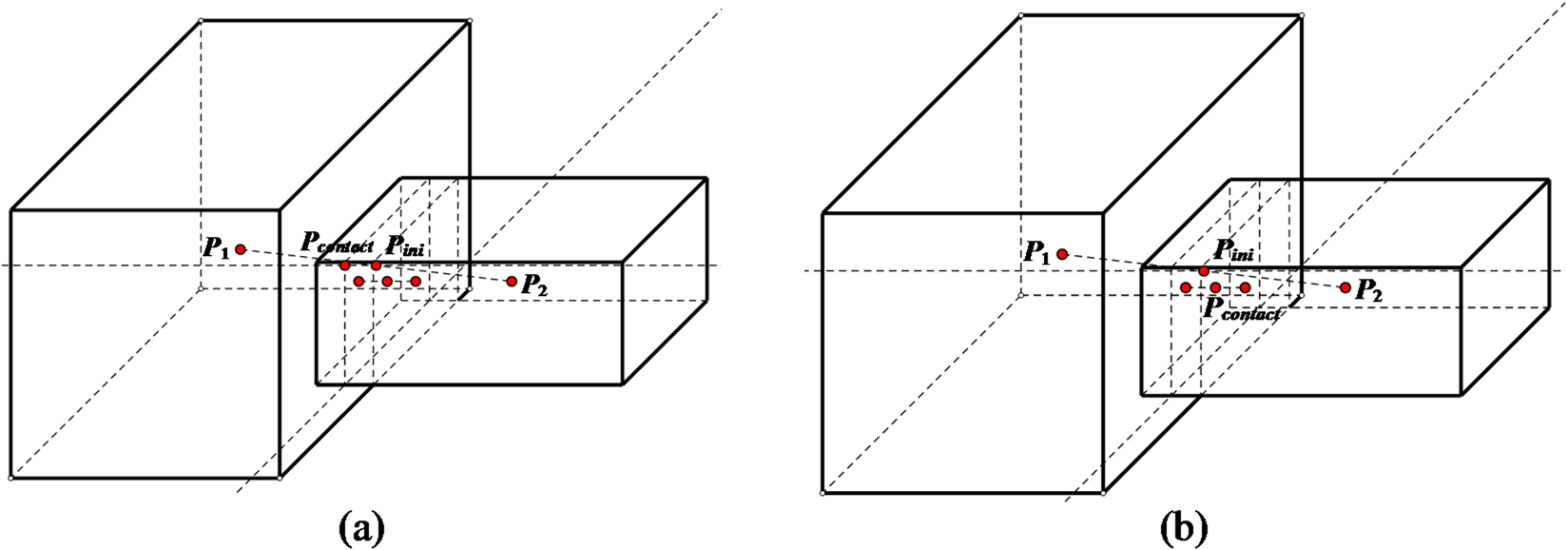
Comparison of updated contact point position in the case of polyhedrons contact. (**a**) Pre-improvement, (**b**) Post-improvement.

At this point, the description of the contact point update algorithm for polyhedral-polyhedral contact is complete. Selecting surface-to-surface contact as the typical contact type for polyhedral-polyhedral contact, the corresponding comparison diagram of the updated contact point positions before and after the improvement is shown in Fig. 9. It can be seen that the updated position of the contact point after improvement is closer to the centroid of the contact area, which is more conducive to contact calculation.

#### Dual-condition termination mechanism

Iteration terminates based on:

(1) **Angle control**: Initial rotation angle = 5°; halved if gap values cease to increase.

(2) **Gap increment threshold**: Termination triggered when incremental gap change falls below a threshold.

#### Improved common-plane algorithm

**Fig. 10:**
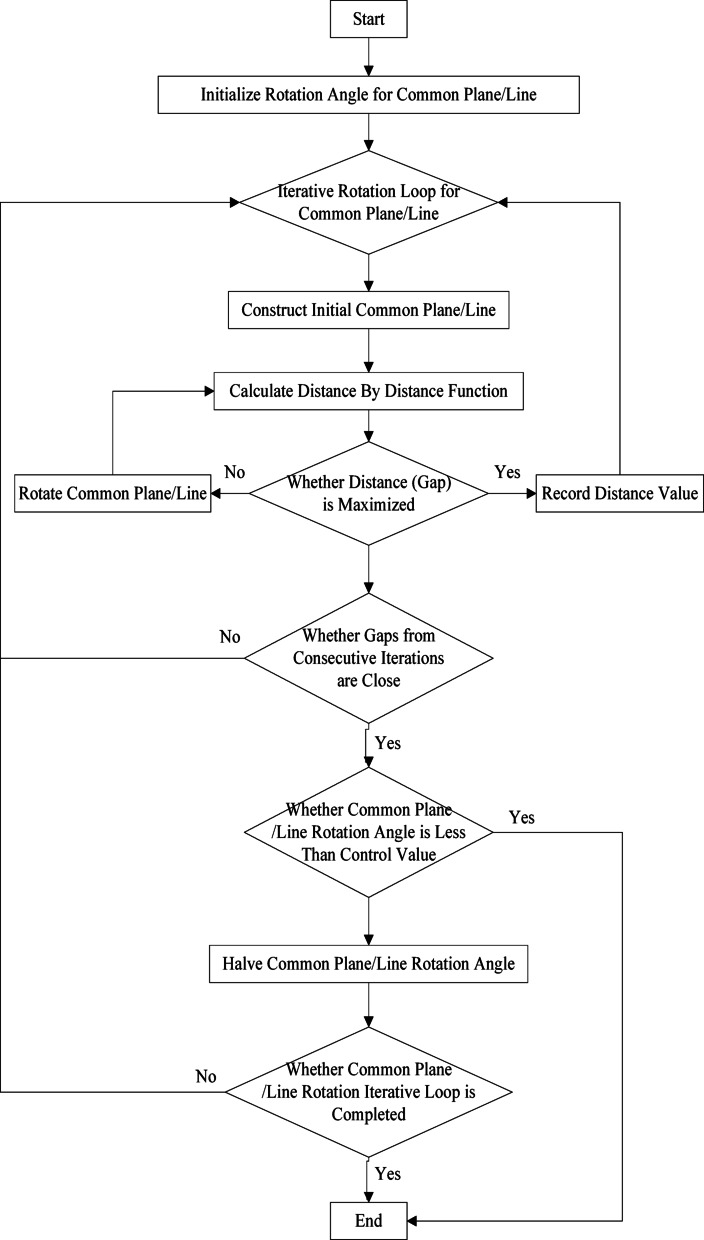
Improved iterative algorithm for the common plane (line) method.

Integrating distance functions, optimized updates, and termination conditions, the enhanced algorithm framework is illustrated in Fig. 10.

## Algorithm validation and performance analysis

### Contact information accuracy

Extract contact points, normal vectors, and depths as reference values through AutoCAD modeling, and compare them with the results obtained from the improved algorithm.

(1) Polygon-polygon contact.

The contact information under polygon-polygon contact condition is shown in Table 2. According to the table, under corner-corner contact condition, the contact point deviation is 24.1966, there is a 0.1092° angle between the contact normals, and the contact depth difference is 0.0001, with an essentially zero error. Under corner-edge contact condition, the contact point deviation is 42.1470, there is a 0° angle between the contact normal, and the contact depth difference is 0, with an error of 0. Under edge-edge contact conditions, the contact point deviation is 118.9070, there is a 0° angle between the contact normal, and the contact depth difference is 0.0001, with an essentially zero error. The maximum and minimum errors for the x-coordinate of the contact points are 12.93% and 0.028%, respectively. The maximum and minimum errors for the y-coordinate of the contact points are 13.21% and 3.10%, respectively. The maximum and minimum errors for the contact normal are 0.078% and 0, respectively. The contact depth error is 0.

**Table 2:** Contact information and relative errors under polygon–polygon contact condition.

Contact condition	Calculation method	Contact Information		
		Contact point (*x*,* y*)	Contact normal (*x*,* y*)	Contact depth/mm
Angle–Angle	CAD calculation (exact value)	(688.5940, 722.2184)	(0.969909,0.243462)	− 57.4266
	Algorithm calculation (value)	(699.532, 699.831)	(0.96991, 0.243462)	− 57.4265
	Absolute difference	(10.938, − 22.3874)	(0.000001,0.0)	0.0001
	Deviation/angle	24.9166	0.1092°	0.0001
	Relative error	(1.59%,3.10%)	0.078%	0
Angle–Edge	CAD Calculation (exact value)	(711.0354, 718.9695)	(0.996317, 0.0857430)	− 22.8660
	Algorithm calculation (value)	(711.234, 761.116)	(0.996318, 0.0857399)	− 22.8660
	Absolute difference	(0.1986,42.1465)	(0.000001, − 0.0000031)	0.0
	Deviation/angle	42.1470	0°	0.0
	Relative error	(0.028%,5.86%)	0	0
Edge–Edge	CAD Calculation (exact value)	(644.0412, 642.5893)	(0.729218, − 0.684278)	− 28.3246
	Algorithm calculation (value)	(727.325, 727.458)	(0.729219, − 0.684281)	− 28.3245
	Absolute difference	(83.2838,84.8687)	(0.000001, − 0.000003)	0.0001
	Deviation/angle	118.9070	0°	0.0001
	Relative error	(12.93%,13.21%)	0	0

The ‘Absolute Difference’ represents the raw deviation between the algorithm-calculated value and the CAD reference value (e.g., Δx = x_alg - x_ref). The ‘Relative Error’ for contact points is calculated as the absolute difference divided by the absolute value of the reference coordinate (e.g., δx = |Δx| / |x_ref|). For contact normals, the relative error is defined as the L2-norm of the difference between the calculated and reference normal vectors (||n_calc - n_ref||_2_). The relative error for contact depth is |Δgap| / |gap_ref|. Table 3 below is also based on Table 2.

(2)Polyhedron-polyhedron contact.

The contact information under polyhedron-polyhedron contact condition is shown in Table 3. According to the table, under face-to-face contact condition, the contact point deviation is 0.2432, there is a 0.07° angle between the contact normal, and the contact depth difference is -1.244901. Under point-to-face contact condition, the contact point deviation is 29.5954, there is a 0.3° angle between the contact normal, and the contact depth difference is -0.6993. Under point-to-point contact condition, the contact point deviation is 4.1460, there is a 0.47° angle between the contact normal, and the contact depth difference is -2.0533. The maximum and minimum errors for the x-coordinate of the contact points are 13.74% and 6.70%, respectively. The maximum and minimum errors for the y-coordinate of the contact points are 8.39% and 0.00012%, respectively. The maximum and minimum errors for the z-coordinate of the contact points are 4.53% and 0, respectively. The maximum and minimum errors for the contact normal are 11.14% and 0.078%, respectively. The maximum and minimum errors for the contact depth are 14.9% and 6.71%, respectively.

**Table 3:** Contact information and relative errors under polyhedron-polyhedron contact condition.

Contact condition	Calculation method	Contact information		
		Contact point (*x*,* y*,*z*)	Contact normal (*x*,* y*,*z*)	Contact depth/mm
Face-face	CAD calculation (exact value)	(1.9658, 344.3764, 21.7791)	(0,− 1,0)	− 8.3549
	Algorithm calculation (value)	(1.72264,344.376,21.7791	(− 0.00128823, − 0.999662,0.0259485)	− 9.60391
	Absolute difference	(− 0.24316, − 0.0004,0.0)	(− 0.00128823,0.000338,0.0259485)	− 1.244901
	Deviation/angle	0.2432	0.07°	− 1.244901
	Relative error	(12.37%,0.00012%,0)	0.078%	14.9%
Point-face	CAD calculation (exact value)	(30.1605, 349.1939,21.7791)	(0.9725, − 0.2327,0.0000)	− 10.4212
	Algorithm calculation (value)	(34.3049,378.481,20.7916)	(0.97879, − 0.239664, − 0.00130589)	− 11.1205
	Absolute difference	(4.1444,29.2871, − 0.9875)	(− 0.00629, − 0.006946, − 0.00130589)	− 0.6993
	Deviation/angle	29.5954	0.3°	− 0.6993
	Relative error	(13.74,8.39%,4.53%)	2.23%	6.71%
Point-point	CAD calculation (exact value)	(28.1974, 349.7175,21.7791)	(0.9973, − 0.0735,0.0000)	− 15.4043
	Algorithm calculation (value)	(26.2242,346.13, 21.1263)	(0.986371, − 0.064451, − 0.00536442)	− 17.4576
	Absolute difference	(− 1.9732, − 3.5874, − 0.6528)	(− 0.010929, 0.009049, − 0.00536442)	− 2.0533
	Deviation/angle	4.1460	0.47°	− 2.0533
	Relative error	(6.70%,1.03%,2.30%)	11.14%	13.33%

Based on the comprehensive analysis of the aforementioned data results, it can be observed that although there are numerical deviations between the contact information obtained through the algorithm and the known contact information, these deviations fall within an acceptable range. Consequently, the contact information derived from the proposed polygon-polygon and polyhedron-polyhedron contact detection algorithms in this paper demonstrates numerical acceptability, thereby validating their correctness.

### DEM simulation and experimental validation

(1) Code verification.

**Fig. 11:**
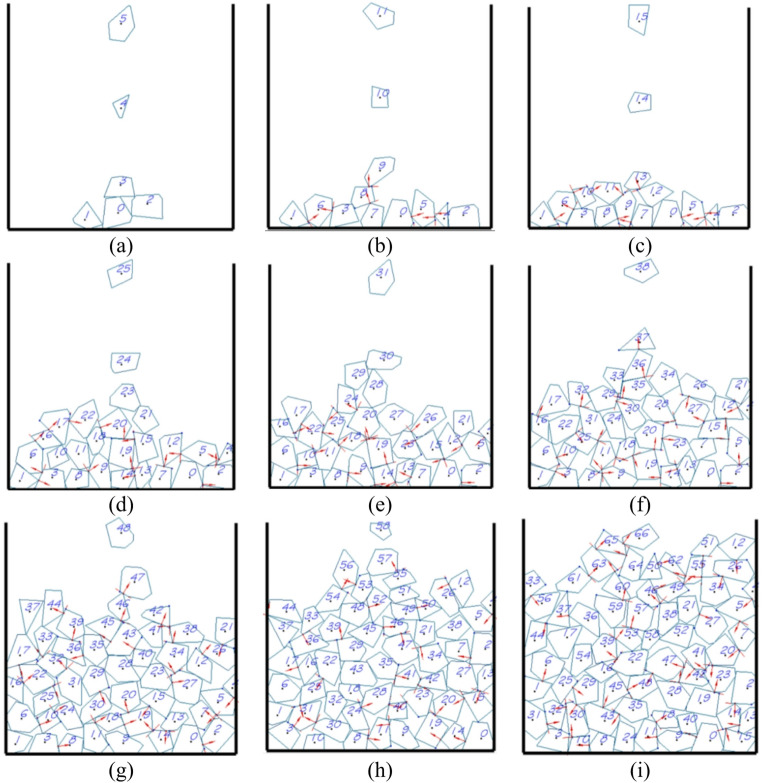
Simulation of random falling and packing process of polygonal elements in a container.

**Fig. 12:**
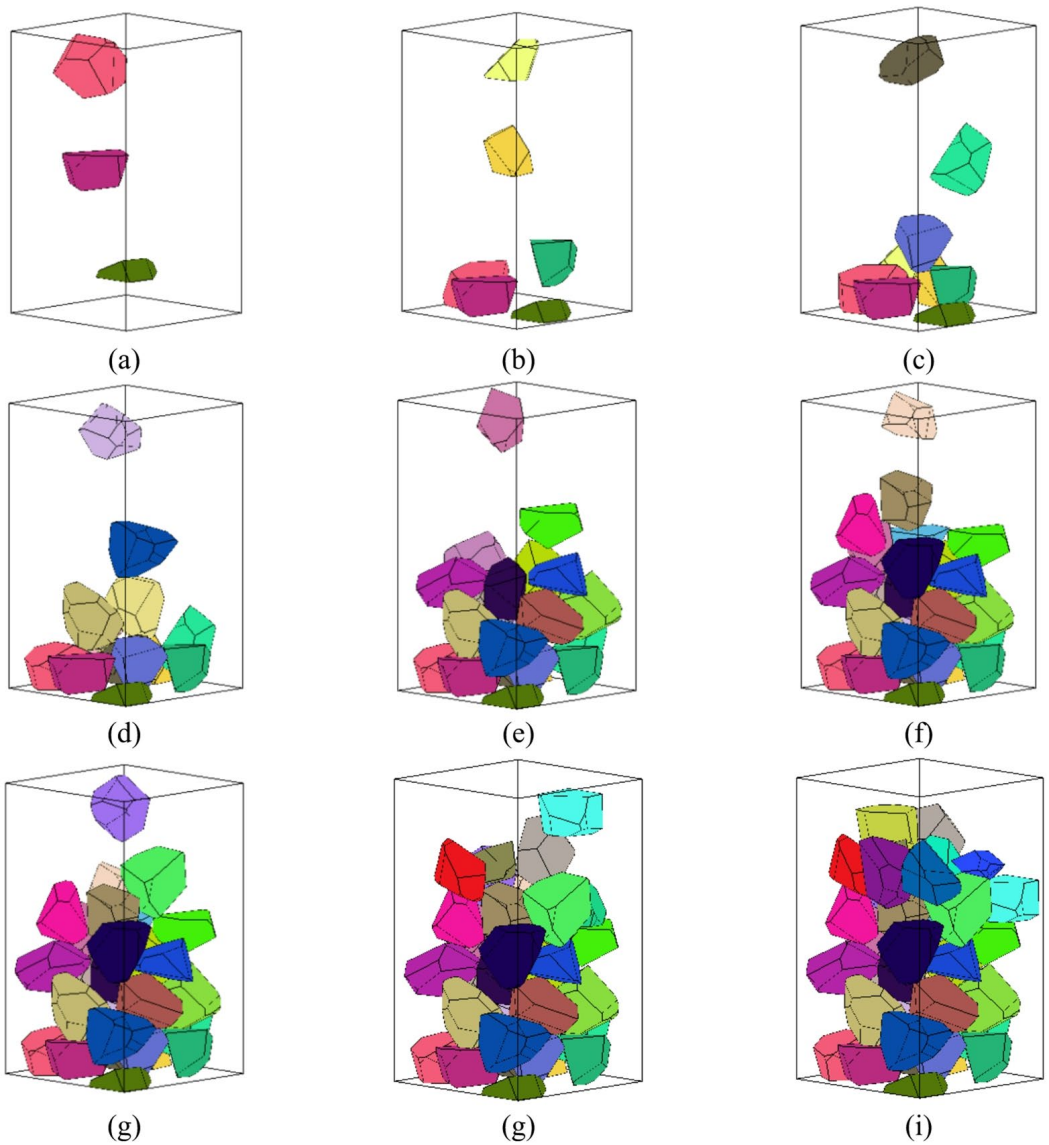
Simulation of random falling and packing process of polyhedral elements in a container.

A DEM (Discrete Element Method) program based on C + + was developed to simulate the random packing process of polygonal and polyhedral elements within a container, as illustrated in Figs. 11 and 12. From the figures, it is evident that the falling patterns of the elements and the resulting packing configurations closely align with real-world scenarios. This validation confirms the reliability of the developed DEM program in simulating the packing process of block elements.

(2)Experimental comparison.

**Fig. 13:**
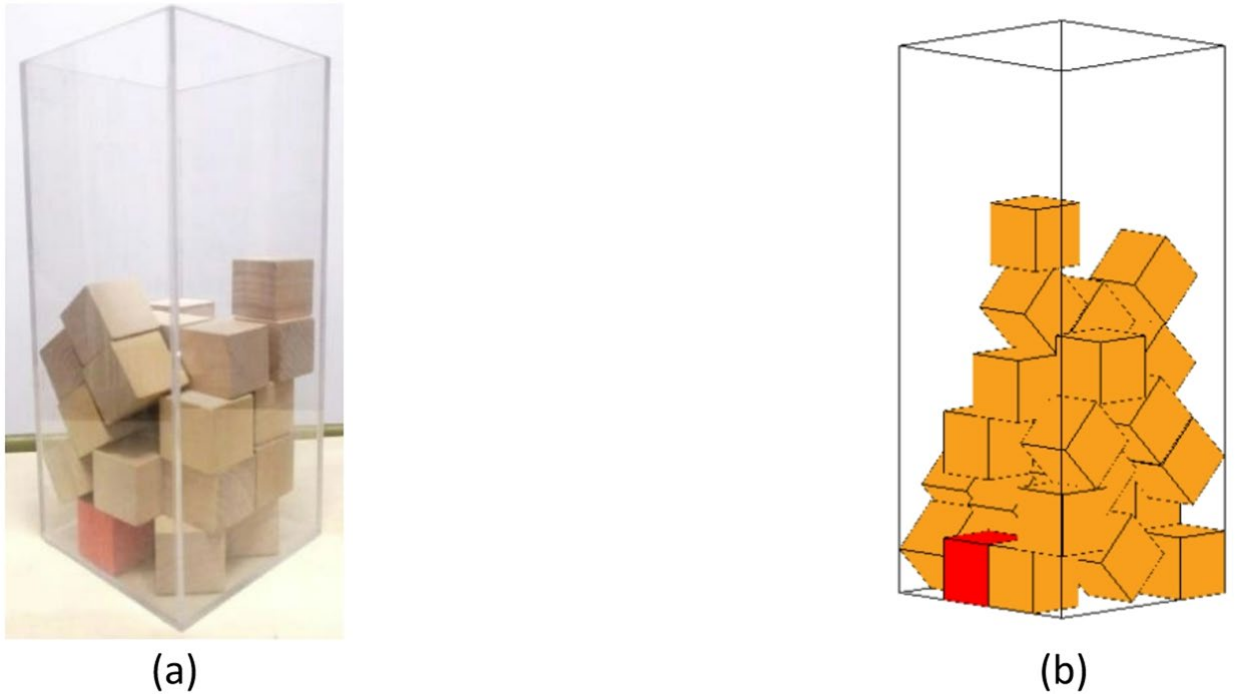
Final position of labeled cubic particles in experiment and simulation during the packing process. (**a**) Experiment, (**b**) Simulation.

In this study, cubes were used as representative particles (block elements) to simulate the particle packing process, with a specific particle marked in red for identification. The final position of the individually marked red cube particle in the experiment is shown in Fig. 13(a), while the corresponding final position of the designated red cube particle in the simulation is depicted in Fig. 13(b).

As observed from the figures above, the final positions of the designated cube particle in both the experiment and simulation exhibit a high degree of similarity, with an angular deviation of approximately 5°. This indicates a strong agreement between the simulated results and the experimental outcomes, validating the accuracy of the Discrete Element Method (DEM) simulation.

**Fig. 14:**
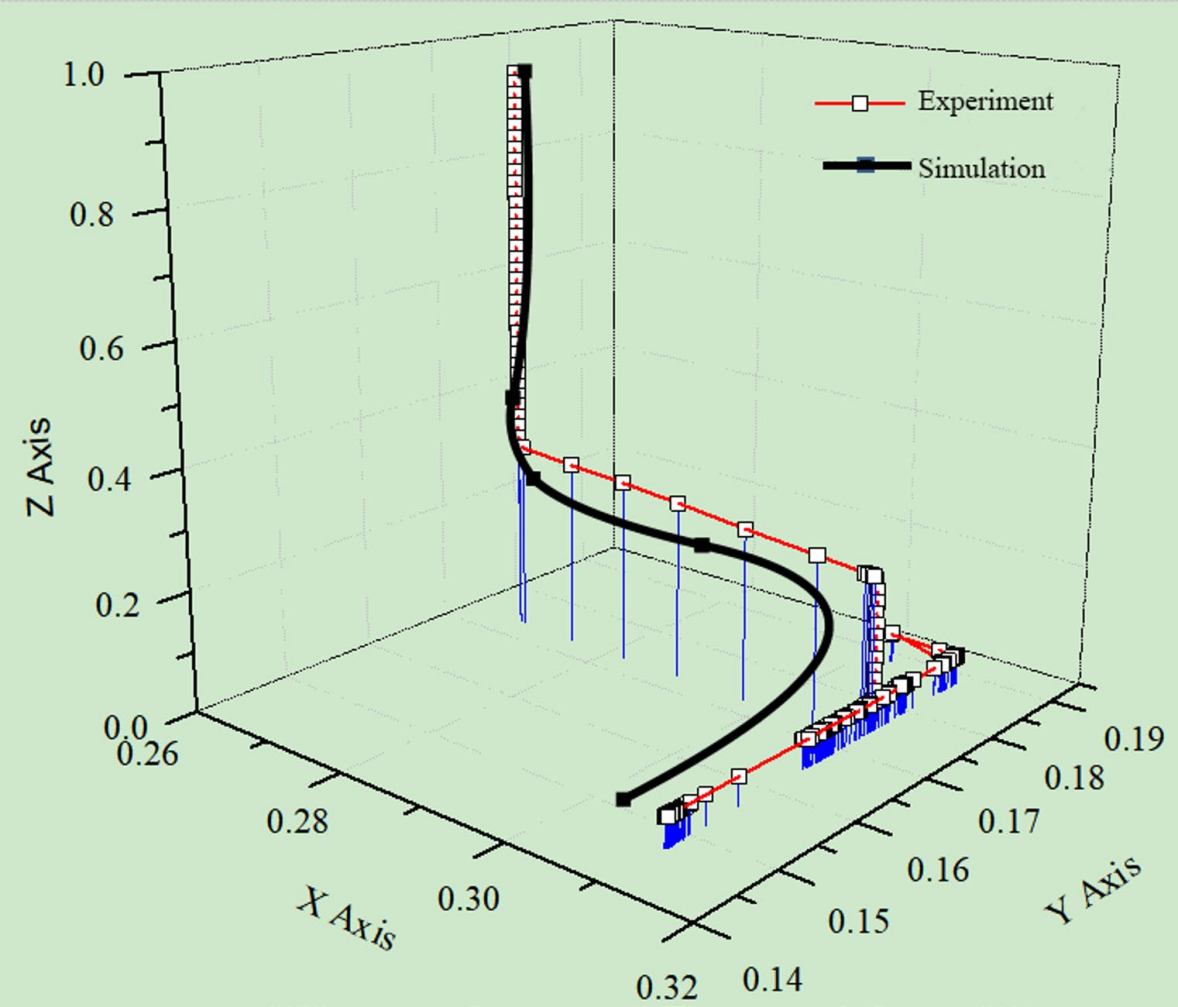
Variation diagram of the centroid coordinates of the specified cubic particles.

The centroid coordinate changes of the designated cube particle, corresponding to Fig. 13, are illustrated in Fig. 14. This figure visualizes the trajectory of the particle from its initial generation, through the falling process, to its final resting position. Notably, the centroid coordinate paths in both the experiment and simulation demonstrate a high level of similarity, further confirming the reliability of the DEM approach in capturing the dynamic behavior of granular particles.

**Fig. 15:**
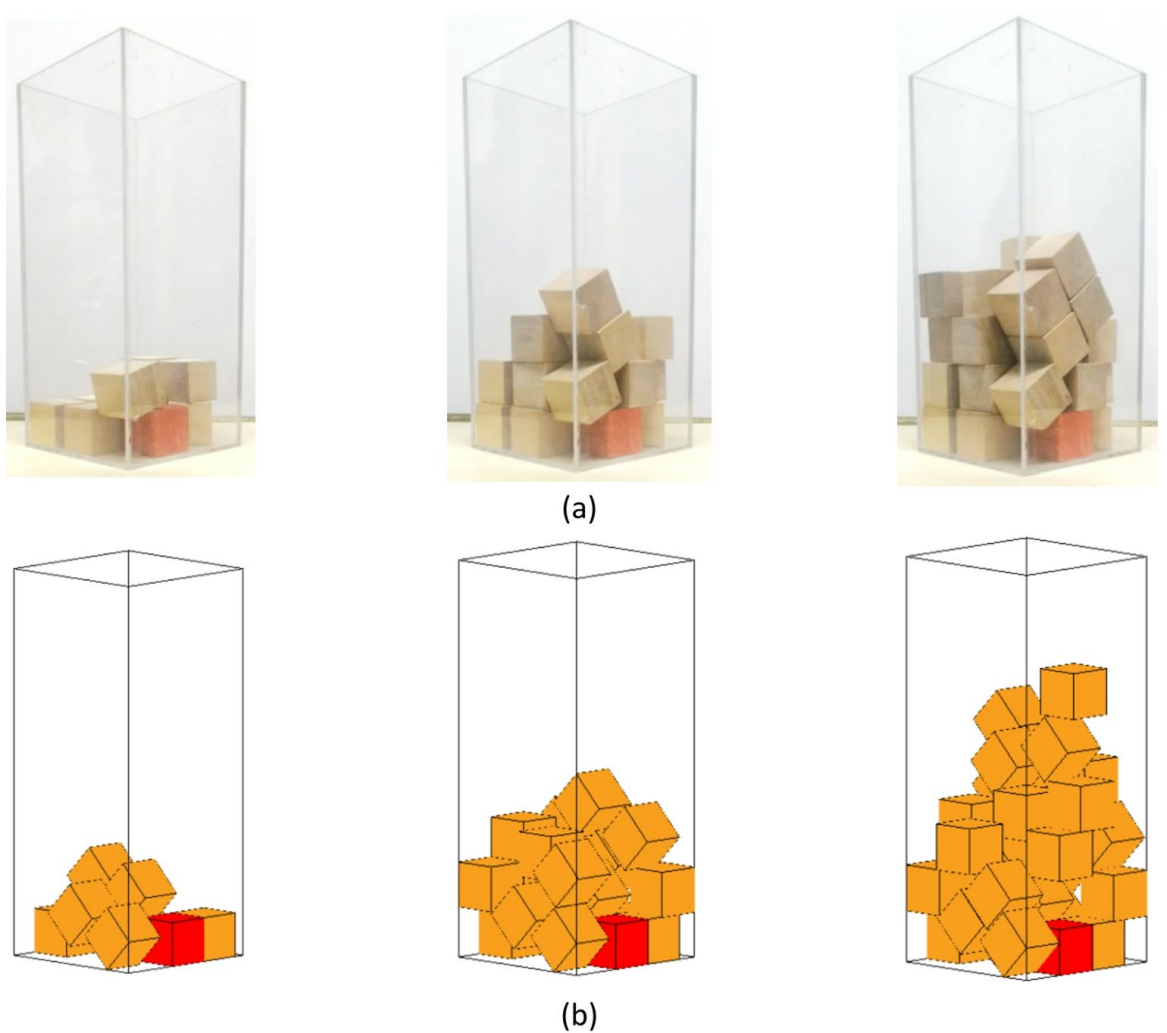
Experiment and simulation of the process of cubical particles stacking in a container. (**a**) Experiment, (**b**) Simulation.

The experiment and simulation of the packing process of cubic particles in a container are shown in Fig. 15. As can be seen from the figure, the morphological changes in the packing of cubic particles in both the experiment and simulation are largely consistent, and the packing height of the cubic particles is relatively close, approximately three-fifths of the container height. The aforementioned analysis results validate the correctness of the proposed contact detection algorithm for polyhedrons in this paper and also demonstrate the reliability of the developed discrete element framework code.

The high consistency between experimental and simulated particle trajectories and final packing morphology (Figs. 13, 14 and 15) validates the overall reliability of the DEM framework and the proposed contact algorithm. More importantly, the improved accuracy in resolving individual contacts (e.g., reduced penetration depth as shown in Tables 4 and 5) directly contributes to a more physically realistic simulation of collective particle behavior. Accurate contact forces are fundamental to correctly predicting macro-mechanical properties such as the angle of repose, stress distribution in silos, and bulk modulus. While this study primarily focuses on the contact detection mechanism itself, the observed reduction in erroneous overlaps (depth error) ensures that the force calculation in the DEM solver is based on more accurate geometric inputs, which is a critical prerequisite for obtaining reliable macro-scale results in future studies focused on specific engineering applications.

### Computational efficiency comparison

All simulations were performed on a workstation equipped with an Intel Core i9-13900 K CPU and 64 GB RAM. The DEM program was compiled using Microsoft Visual Studio 2022 with O2 optimization enabled.

(1)Polygon-polygon.

Table 4 presents the numerical values of computational time, maximum iteration number, element count, and maximum contact depth obtained from simulations of the polygonal element packing process in a container.

**Table 4:** Relevant numerical values obtained from packing simulation of polygonal elements using two different algorithms.

Algorithm name	Computational time	Maximum iterations	Number of elements	Maximum contact depth (m)
Cundall’s Common plane method	26,342s	22	137	− 0.00327
Improved common plane method	23,536s	4	140	− 0.00152
Difference	2806s	18	3	− 0.00175
speedup ratio	10.65%	81.82%	2.17%	53.52%

As shown in the table, under identical simulation conditions, compared with Cundall’s common plane method, the polygon-polygon contact detection algorithm proposed in this study based on the improved common plane method requires less computational time, achieving a 10.65% increase in computational efficiency. This improvement stems from the modified termination criteria in the iterative process of the original Cundall method, which reduces the iteration steps by 81.82% during polygon-polygon contact detection. Furthermore, the maximum contact depth produced by the proposed algorithm is smaller than that of the common plane method, demonstrating a 53.52% improvement in precision, which is more conducive to contact calculations involving polygonal elements.

**Fig. 16:**
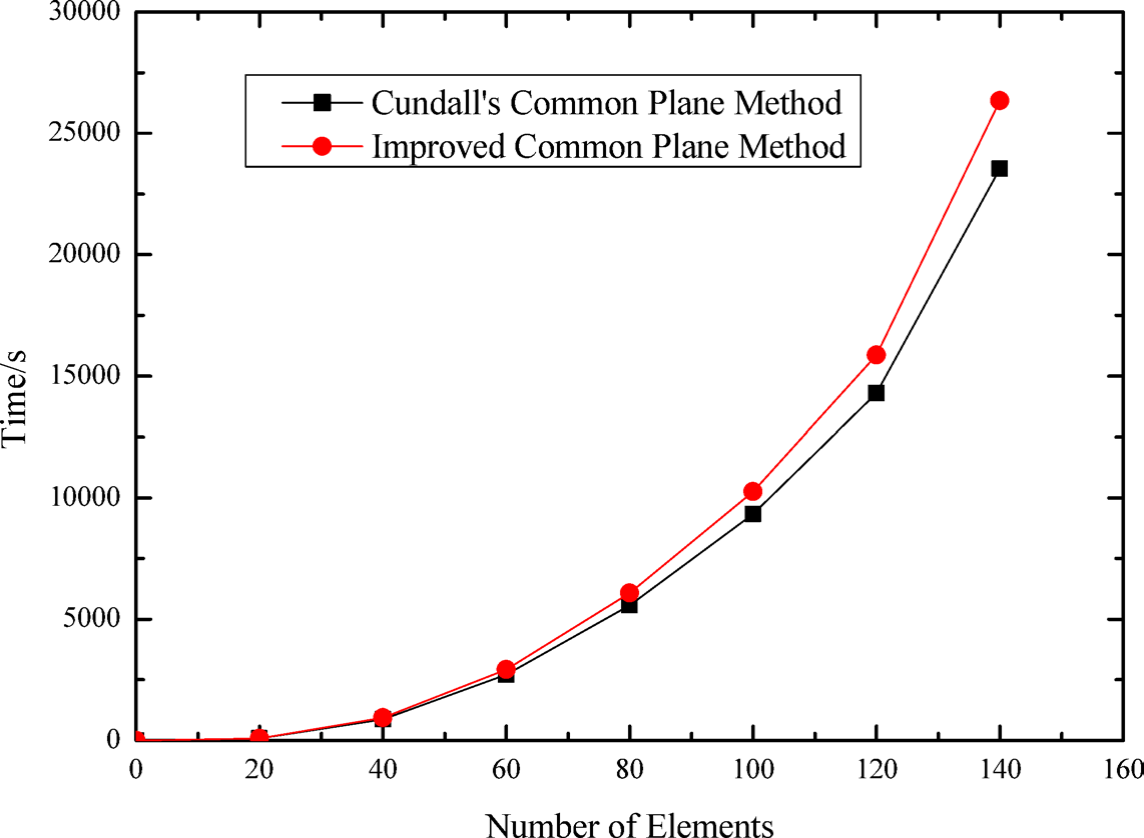
Relationship curves between polygonal element count and computation time.

**Fig. 17:**
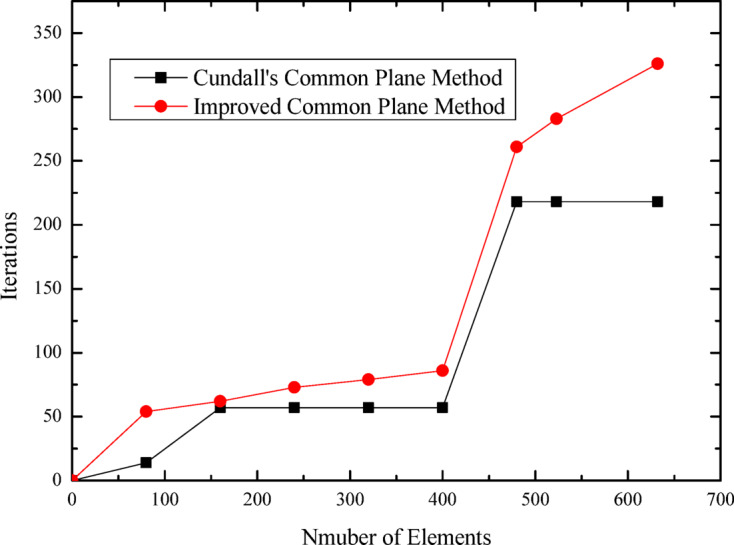
Relationship curves between polygonal element count and number of iterations.

The relationship curves between the number of polygonal elements and computational time, as well as between element count and iteration steps, are presented in Figs. 16 and 17, respectively. These figures illustrate the specific variation processes of computational time and iteration steps with increasing element numbers. It can be observed that for the same number of elements, the proposed algorithm based on the improved common plane method exhibits significantly lower computational time and fewer iteration steps compared to Cundall’s method.

(2) Polyhedron-polyhedron.

Table 5 presents the numerical results of computational time, maximum iterations, number of elements, and maximum contact depth obtained from the simulation of polyhedral element packing process in a container.

**Table 5:** Relevant numerical values obtained from packing simulation of polyhedral elements using two different algorithms.

Algorithm name	Computational time	Maximum iterations	Number of elements	Maximum contact depth (m)
Cundall’s Common plane method	2292456s	326	626	− 0.0025463
Improved common plane method	2163475s	218	632	− 0.0013397
Difference	128981s	108	6	− 0.0012066
speedup ratio	5.63%	33.13%	0.9%	47.39%

As indicated in the table, compared with Cundall’s common plane method, the polyhedron-polyhedron contact detection algorithm proposed in this study based on the improved common plane method reduces simulation computation time, achieving a 5.63% increase in computational efficiency. Under similar element counts, this algorithm also reduces iteration steps by 33.13% during contact detection. Furthermore, the proposed algorithm yields smaller contact depths with a 47.39% improvement in precision, which is more beneficial for contact calculations involving polyhedral elements.

**Fig. 18:**
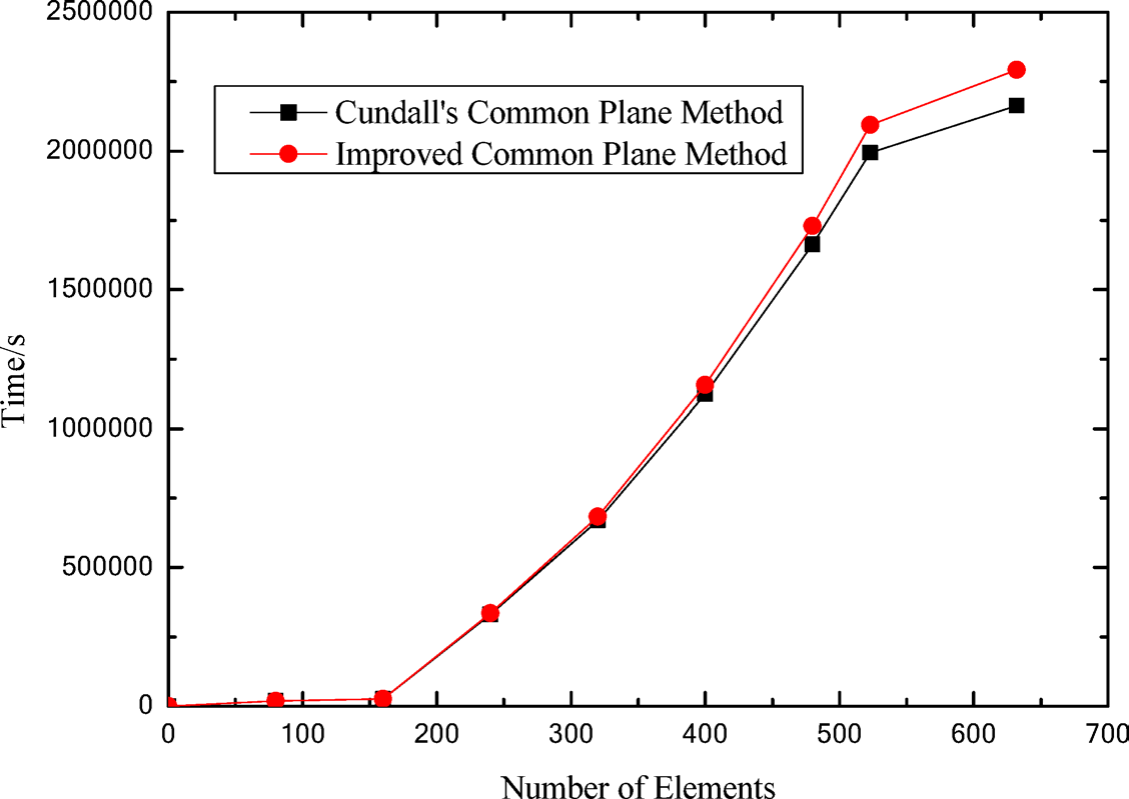
Relationship curves between polyhedral element count and computation time.

**Fig. 19:**
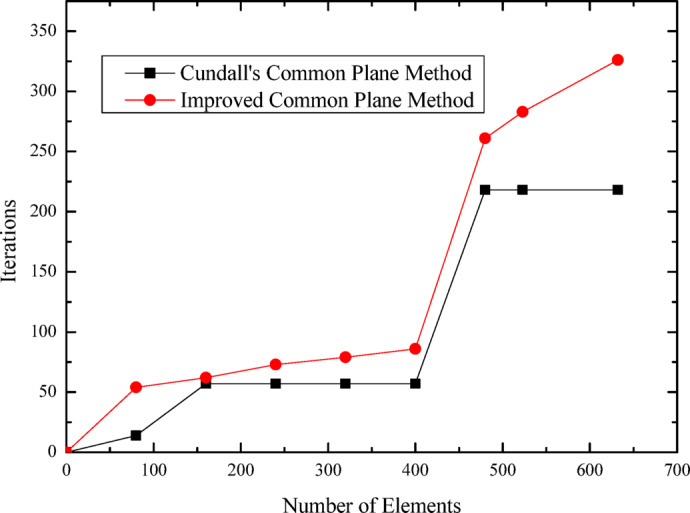
Relationship curves between polyhedral element count and number of iterations.

The relationship curves between polyhedral element count and computation time, as well as between element count and iteration steps, are presented in Figs. 18 and 19, respectively. These figures illustrate the specific variation processes of computation time and iteration steps with increasing element numbers. It can be observed that for the same number of elements, the proposed algorithm based on the improved common plane method demonstrates lower computation time and fewer iteration steps compared to Cundall’s method.

The primary baseline for comparison in this study is the traditional Cundall’s CPM, as our work is a direct improvement upon it. A comprehensive comparative analysis against other state-of-the-art algorithms (e.g., GJK-EPA, SAT) under identical tolerances and hardware conditions, measuring accuracy, time, iterations, and failure rates, is an important area for future research. Such a comparison would provide a more complete picture of the proposed algorithm’s standing in the field. Nonetheless, the significant reduction in iteration count (Figs. 17 and 19) directly translates to a lower per-contact computational cost, which is a key factor in the observed overall speedup. The algorithm’s time complexity per iteration is linear with respect to the number of vertices of the involved polyhedrons, O(N_v), for the distance function calculation. The overall scalability with respect to the number of contacts (N_c) is approximately O(N_c * K), where K is the average number of iterations per contact, which our method significantly reduces.

## Discussion and limitations

While the improved common-plane algorithm demonstrates enhanced accuracy and efficiency in the tested scenarios, it is essential to acknowledge its limitations to guide future research and application.

1. **Computational Overhead for Irregular Geometries**: The algorithm’s performance is optimal for polyhedrons with a moderate number of faces and vertices. For highly irregular polyhedrons with numerous faces or non-uniform tessellation, the computation of distance functions and the projection operations may introduce increased overhead, potentially diminishing the efficiency gains compared to simpler bodies.
2. **Scalability in Large-Scale Simulations**: Although the improved iteration reduces steps per contact, the algorithm currently operates as a precise detection method without an integrated broad-phase (e.g., spatial partitioning like BVH). In simulations involving hundreds of thousands of elements, the absence of a hierarchical culling mechanism could become a bottleneck. Future work will integrate spatial indexing structures to address this scalability issue.
3. **Tolerance Policy and Degenerate Cases**: The algorithm employs a distance tolerance for determining contact states and an angular tolerance (as part of the dual-condition termination) to handle near-coplanar faces and near-parallel edges. Stress tests involving these degenerate cases (e.g., coplanar faces with a gap near the floating-point precision, very short edges) were conducted. The dual-condition termination mechanism proved robust in preventing infinite loops. However, the accuracy of contact point resolution in such extreme scenarios is highly sensitive to the chosen tolerances, which were set empirically based on the typical element size in our simulations.
4. **Convexity Assumption**: The current algorithm is designed for convex polyhedrons. Non-convex particles must be decomposed into convex constituents beforehand, and contacts are detected between these convex parts. The “max on one body, min on the other” distance calculation is inherent to the common-plane concept for convex shapes and ensures a unique separation plane, avoiding bias in symmetric contacts as it is a geometric property of the two bodies rather than an arbitrary choice.

## Conclusions

1. The improved common-plane algorithm enhances computational efficiency and accuracy by transforming contact detection into block-to-plane distance problems.
2. Optimized contact point updates and dual-condition termination mitigate traditional CPM flaws.
3. Experimental and simulation results confirm the algorithm’s reliability, with particle trajectories and packing morphologies closely matching.
4. Compared to Cundall’s method, the algorithm achieves higher efficiency, fewer iterations, and smaller contact depths, advancing DEM applications for complex-shaped elements.

## Data Availability

Data is provided within the manuscript .

## References

[1] Cundall, P. A. A computer model for simulating progressive large-scale movement in blocky rock system[C]. In *Proceedings of the International Symposium on Rock Mechanics* **8**, 129–136 (1971).

[2] Cundall, P. A. & Strack, O. D. L. A discrete numerical model for granular assemblies[J]. *Geotechnique* **29** (1), 47–65. 10.1680/geot.1979.29.1.47 (1979).

[3] Cundall, P. A. Formulation of a three-dimensional distinct element model—Part I. A scheme to detect and represent contacts in a system composed of many polyhedral blocks[J]. *Int. J. Rock. Mech. Min. Sci. Geomech. Abstracts Pergamon*. **25** (3), 107–116. 10.1016/0148-9062(88)92293-0 (1988).

[4] Hart, R., Cundall, P. A. & Lemos, J. Formulation of a three-dimensional distinct element model—Part II. Mechanical calculations for motion and interaction of a system composed of many polyhedral blocks[J]. *Int. J. Rock. Mech. Min. Sci. Geomech. Abstracts Pergamon*. **25** (3), 117–125. 10.1016/0148-9062(88)92294-2 (1988).

[5] Al-Hashemi, H. M. B. & Al-Amoudi, O. S. B. A review on the angle of repose of granular materials[J]. *Powder Technol.* **330**, 397–417. 10.1016/j.powtec.2018.02.003 (2018).

[6] Ulusoy, U. A review of particle shape effects on material properties for various engineering applications: from macro to nanoscale[J]. *Minerals* **13** (1), 91. 10.3390/min13010091 (2023).

[7] Wang, X. et al. Developments and applications of the CFD-DEM method in particle–fluid numerical simulation in petroleum engineering: A review[J]. *Appl. Therm. Eng.* **222**, 119865. 10.1016/j.applthermaleng.2022.119865 (2023).

[8] Li, R., Duan, G. & Sakai, M. DEM simulations in nuclear engineering: a review of recent progress[J]. *J. Nucl. Sci. Technol.* **61** (3), 285–306. 10.1080/00223131.2023.2231969 (2024).

[9] Xu, L., Bao, S. & Zhao, Y. Multi-level DEM study on liner wear in tumbling mills for an engineering level approach[J]. *Powder Technol.* **364**, 332–342. 10.1016/j.powtec.2020.02.004 (2020).

[10] Song, X. et al. Calibration of DEM models for fertilizer particles based on numerical simulations and granular experiments[J]. *Comput. Electron. Agric.* **204**, 107507. 10.1016/j.compag.2022.107507 (2023).

[11] Nie, Y. et al. DEM study on the macro-and micromechanical behaviours of breakable granular materials under Cyclic loading[J]. *Transp. Geotechnics*. **38**, 100915. 10.1016/j.trgeo.2022.100915 (2023).

[12] Qi, Q. et al. Investigation of the compaction behaviour of sand-gravel mixtures via DEM: effect of the sand particle shape under vibration loading[J]. *Comput. Geotech.* **154**, 105153. 10.1016/j.compgeo.2022.105153 (2023).

[13] Descantes, Y., Tricoire, F. & Richard, P. Classical contact detection algorithms for 3D DEM simulations: drawbacks and solutions[J]. *Comput. Geotech.* **114**, 103134. 10.1016/j.compgeo.2019.103134 (2019).

[14] Huang, P. et al. An improved contact detection algorithm for bonded particles based on multi-level grid and bounding box in DEM simulation[J]. *Powder Technol.* **374**, 577–596. 10.1016/j.powtec.2020.07.022 (2020).

[15] Zhou, Q., Xu, W. J. & Liu, G. Y. A contact detection algorithm for triangle boundary in GPU-based DEM and its application in a large-scale landslide[J]. *Comput. Geotech.* **138**, 104371. 10.1016/j.compgeo.2021.104371 (2021).

[16] Tian, Y., Zeng, Z. & Xing, Y. A review of discrete element method applications in Soil–Plant interactions: challenges and Opportunities[J]. *Agriculture* **14** (9), 1486. 10.3390/agriculture14091486 (2024).

[17] Zhang, Y. *Development of Modeling Techniques Using Discrete Elements with Applications To Civil Engineering Systems[M]* (University of Florida, 2020).

[18] Boon, C. W., Houlsby, G. T. & Utili, S. A new algorithm for contact detection between convex polygonal and polyhedral particles in the discrete element method[J]. *Comput. Geotech.* **44**, 73–82. 10.1016/j.compgeo.2012.03.012 (2012).

[19] Toe, D. et al. A novel DEM approach to simulate block propagation on forested slopes[J]. *Rock Mech. Rock Eng.* **51**, 811–825. 10.1007/s00603-017-1348-2 (2018).

[20] Lu, R. et al. Comparison of clumps and rigid blocks in three-dimensional DEM simulations: curvature-based shape characterization[J]. *Comput. Geotech.* **151**, 104991. 10.1016/j.compgeo.2022.104991 (2022).

[21] Kassotakis, N. & Sarhosis, V. *Employing non-contact Sensing Techniques for Improving Efficiency and Automation in Numerical Modelling of Existing Masonry Structures: A Critical Literature review[C]//Structures* Vol. 32, 1777–1797 (Elsevier, 2021). 10.1016/j.istruc.2021.03.111.

[22] Lai, Z., Chen, Q. & Huang, L. Machine-learning‐enabled discrete element method: contact detection and resolution of irregular‐shaped particles[J]. *Int. J. Numer. Anal. Meth. Geomech.* **46** (1), 113–140. 10.1002/nag.3293 (2022).

[23] Neto, A. G. Framework for automatic contact detection in a multibody system[J]. *Comput. Methods Appl. Mech. Eng.* **403**, 115703. 10.1016/j.cma.2022.115703 (2023).

